# Lessons learnt from the process of designing care coordination interventions through participatory action research in public healthcare networks of six Latin American countries

**DOI:** 10.1186/s12961-023-00985-9

**Published:** 2023-06-01

**Authors:** Ingrid Vargas, Amparo-Susana Mogollón-Pérez, Pamela Eguiguren, Isabella Samico, Fernando Bertolotto, Julieta López-Vázquez, Delia-Inés Amarilla, Pierre De Paepe, María-Luisa Vázquez, for Equity-LA II, Julia Puzzolo, Marisel Colautti, Alicia Aronna, Irene Luppi, Cecilia Muruaga, Francisco Leone, Mario Rovere, Adriana Huerta, Claudio Alonso, Héctor Hoet, María Porpatto, Elba Hernández, María Inés Stapaj, Fernando Vignone, Leonardo Caruana, Marina Mendes, Cecylia Oliveira, Hylany Almeida, Renata Freitas, Cynthia Resque, Geison Silva, Luciana Dubeux, Isabel Guzmán, Patricio Alvarez, Ana-María Oyarce, Andrea Alvarez, Nimsi Pastén, Viviana Rojas, Paola González, Jorge Caro, Isabel Abarca, Maria Eugenia Chadwick, Patricia Espejo, Mauricio Araya, Wilma Hidalgo ySergio Rojas, Virginia Garcia, Angela-María Pinzón, Heisel-Gloria León, Andrés Gallego, Carol Cardoso, Laura Bejarano, Josefina Chávez, Silvia Ballesteros, Leonardo Gómez, Cesar Santamaría, Carmen Villamizar, Amanda Salinas, Cristian Cortés, Carolina Larrañaga, Haidy Carolina Rivera, Edgar Fabián Sanabria, Omar Velandia, Carlos Solórzano, Angélica-Ivonne Cisneros, Edit Rodríguez, Damián-Eduardo Pérez, Vianey González, Néstor-Iván Cabrera, Daniel Córdoba, Sebastián Gadea, Camila Estiben, Luciana Piccardo, Graciela García, Cecilia Acosta, María-Noel Ballarini

**Affiliations:** 1 Health Policy and Health Services Research Group, Health Policy Research Unit, Consortium for Health Care and Social Services of Catalonia, Avenida Tibidabo 21, 08022 Barcelona, Spain; 2 Escuela de Medicina y Ciencias de la Salud, Universidad del Rosario, Cra 24 No. 63C-69, Quinta Mutis, 11001 Bogotá, Colombia; 3 Escuela de Salud Pública Dr. Salvador Allende Gossens, Facultad de Medicina, Universidad de Chile, Avenida Independencia, 939 Santiago de Chile, Chile; 4 Instituto de Medicina Integral Prof. Fernando Figueira-IMIP, Rua Dos Coelhos No. 300, Boa Vista, 50070-550 Recife, Brasil; 5 Facultad de Enfermería, Universidad de la República, Avenida 18 de Julio 124, 11200 Montevideo, Uruguay; 6 Instituto de Salud Pública, Universidad Veracruzana, Avenida Dr. Luis Castelazo Ayala S/N. Col. Industrial Ánimas, 91190 Xalapa, Veracruz México; 7 Centro de Estudios Interdisciplinarios, Universidad Nacional de Rosario, Rosario, Argentina; 8 Public Sector Care Unit, Department of Public Health, Prince Leopold Institute of Tropical Medicine, Nationalestraat 155, 2000 Antwerp, Belgium

**Keywords:** Care coordination, Integrated delivery systems, Participatory action research, Health services research, Qualitative research, Implementation science, Diffusion of innovation, Latin America

## Abstract

**Background:**

The participation of health professionals in designing interventions is considered vital to effective implementation, yet in areas such as clinical coordination is rarely promoted and evaluated. This study, part of Equity-LA II, aims to analyse the design process of interventions to improve clinical coordination, taking a participatory-action-research (PAR) approach, in healthcare networks of Argentina, Brazil, Chile, Colombia, Mexico and Uruguay. This participatory process was planned in four phases, led by a local steering committee (LSC): (1) dissemination of problem analysis results and creation of professionals’ platform, (2) selection of problems and intervention (3) intervention design and planning (4) adjustments after evaluation of first implementation stage.

**Methods:**

A descriptive qualitative study based on documentary analysis, using a topic guide, was conducted in each intervention network. Documents produced regarding the intervention design process were selected. Thematic content analysis was conducted, generating mixed categories taken from the topic guide and identified from data. Main categories were LSC characteristics, type of design process (phases, participants’ roles, methods) and associated difficulties, coordination problems and interventions selected.

**Results:**

LSCs of similar composition (managers, professionals and researchers) were established, with increasing membership in Chile and high turnover in Argentina, Colombia and Mexico. Following results dissemination and selection of problems and interventions (more participatory in Chile and Colombia: 200–479 participants), the interventions were designed and planned, resulting in three different types of processes: (1) short initial design with adjustments after first implementation stage, in Colombia, Brazil and Mexico; (2) longer, more participatory process, with multiple cycles of action/reflection and pilot tests, in Chile; (3) open-ended design for ongoing adaptation, in Argentina and Uruguay. Professionals’ time and the political cycle were the main barriers to participation. The clinical coordination problem selected was limited communication between primary and secondary care doctors. To address it, through discussions guided by context and feasibility criteria, interventions based on mutual feedback were selected.

**Conclusions:**

As expected in a flexible PAR process, its rollout differed across countries in participation and PAR cycles. Results show that PAR can help to design interventions adapted to context and offers lessons that can be applied in other contexts.

## Background

Poor clinical coordination across care levels in healthcare networks is considered to be one of the main obstacles to providing quality of care in many healthcare systems around the world, including Latin America, leading to duplication of diagnostic tests, delays and inconsistencies in treatment, or inappropriate referrals [1–3]. Concerns over poor clinical coordination have sparked the introduction of a variety of clinical coordination mechanisms [4–6]: (1) based on programming, useful for those situations which can be anticipated—and therefore standardized—and do not necessarily require a rapid response (e.g. clinical guidelines); (2) based on feedback between individuals in order to solve the problem at the same level at which the information was generated, useful when the volume of information to be processed is high and the activities are highly specialized and interdependent (e.g. liaison positions or multidisciplinary cross-level teams). Mechanisms to improve clinical coordination—defined as the harmonious connection of the different services needed to provide care to a patient throughout the care continuum in order to achieve a common objective without conflicts [7]—can play an important role in improving quality of care due to their potential to improve the transfer of clinical information and the communication needed to coordinate activities between providers (information coordination), and the provision of care in a sequential and complementary way (clinical management coordination) [8, 9].

However, studies in the region show low levels of adoption of the coordination mechanisms introduced, and point toward the limited adaptation and dissemination of mechanisms to the local context as possible causes [10–12]. For this reason, it is increasingly argued in various fields and disciplines that the different stakeholders have a crucial collaborative role to play in the intervention design process in order to take local priorities into account and achieve better outcomes. This collaboration may take a consultative form, as in knowledge translation models [13], in which the stakeholders give their opinion to tailor evidence-based interventions to the context [14], or may be more participatory in nature [15, 16], with researchers and professionals co-producing or co-designing the intervention [14]. Within this second category, one of the most commonly used approaches in the field of public health is participatory action research (PAR), particularly in the design of community-based interventions [17], and also, although to a much lesser degree, in health services interventions aimed at health professionals [18–20].

### PAR and its benefits for the design and implementation of health services interventions to improve care coordination.

The main features of participatory action research are [18]: (1) repeated cycles of planning, action and evaluation, in which lessons learnt from the action phase form the starting point for the next cycle; (2) collaboration between the researchers and local people or practitioners; and (3) participation and democracy in the different phases of the research project, i.e. the research subjects play an active role, decisions are made together and knowledge is built collectively, so the project is not entirely in the hands of the research team. These characteristics can be present to varying degrees in a research project, and even fluctuate over the course of the process.

In terms of participation, the most participatory types of PAR are cooperative models, in which locals and outside researchers work together but the researchers are in charge of the process, and co-learning models, in which the locals and researchers share knowledge, there is mutual learning, and the researchers act as facilitators [15, 18].

Due to the problem-focused, context-specific and action-oriented [18] nature of the PAR approach and stakeholder involvement, PAR can bring significant benefits to the design and implementation of policies and interventions in health services, particularly those aimed at improving coordination between care levels: (1) it can make the designed intervention more relevant, credible and socially valid, in the sense that it helps produce strategies to solve the problems that professionals face in their daily practice [18]; (2) it boosts the uptake of the intervention, as it makes professionals more likely to trust, make use of and respond positively to the measures proposed and the changes involved when they are included in decisions made on the services that affect their daily practice [21]; (3) it encourages the creation of spaces that foster communication and respect between the actors involved, a key factor in the implementation of strategies to improve cross-level coordination; (4) it helps to disseminate the strategies implemented throughout the organization in a more efficient way [22].

### What should a participatory design for health services interventions look like?

The design of an intervention through a PAR approach is a reflexive, flexible and iterative process that encompasses the identification of problems, selection and design of interventions, and adjustments [16]. It has two key features: first, the initial design of the intervention can be modified over the course of time (some authors refer to this as evolutionary or emergent design) [18, 21]; second, the design, planning and implementation of the intervention are interwoven, so it is often difficult to separate or distinguish one phase or step from another [18]. Systematic documentation of the process and changes that take place throughout is key in applying the cyclical method of planning-action-evaluation, and in the co-creation of knowledge [21, 23]. Thus the documents produced during the process provide relevant evidence and are highly useful for obtaining the full picture of the process [24].

Some authors [18] have made recommendations on executing this type of design process, such as the establishment of clearly defined objectives and phases, the use of suitable strategies to select and retain participants, rigorous knowledge production and documentation of the entire process, taking the influence of the local context into account in intervention planning and implementation, and ensuring an adequate timeframe for the project, with the completion of at least two full PAR cycles to investigate and effect changes with the intervention [18]. However, these recommendations are still of a general nature and have little basis in systematic analysis of the evidence, as evaluations of participatory intervention designs are scarce [18]. Thus little is known about concrete operational aspects such as selection of strategies, the mechanisms of participation or the most suitable collaboration models for a participatory design [22].

Moreover, in the specific domain of cross-level clinical coordination, the use of PAR for the design of interventions is very limited [25], which poses many unknowns, such as how best to carry out a participatory process with health professionals of different care levels who normally work independently of each other, what kind of difficulties arise in these process, or what type of problems and interventions may result from this approach.

### Equity-LA II and the participatory process for the design of the interventions

This study is part of a wider implementation research project (Equity-LA II) [26] that aimed to evaluate the effectiveness of interventions—designed and implemented through PAR processes—in improving clinical coordination and continuity between care levels in healthcare networks of the public healthcare subsystem of Argentina, Brazil, Chile, Colombia, Mexico and Uruguay. Equity-LA II adopted a quasi-experimental design (a controlled before and after design) [27, 28] with an intervention and a control healthcare network in each LA country, combining qualitative research methods to evaluate the intervention design and implementation process with quantitative methods to analyse their effectiveness (after 18 months’ implementation). The study healthcare networks were selected according to the following criteria: (a) provision of a continuum of services to a defined population, including at least primary and secondary care; (b) mainly in urban areas of low or medium–low socioeconomic status; and (c) willingness to participate. Primary care is the entry point to the healthcare network and coordinator of patient care [29]. The most frequently used clinical coordination mechanism between levels of care is the referral form. Previous studies show limited implementation of other information or clinical management coordination mechanisms between care levels [10, 30]. The results of the qualitative study on the context and process factors that influenced the implementation of the interventions, and of another on the impact of PAR interventions on care coordination across levels from the participants’ viewpoint, have already been published [31, 32], as have the results of the quantitative evaluation of the effectiveness of the interventions in terms of care coordination [33] and continuity [34].

The focus of this paper, which complements the previous publications [31–34], is the analysis of the intervention’s design process in itself, which is considered key to understanding the planning process and content-related elements that may have influenced the intervention outcomes [13], as well as helping us to draw lessons for its replication in other contexts.

#### The participatory process for the design of the interventions

Within the framework of a reflexive and flexible process, some general elements were agreed upon by the different Equity-LA II research teams (from six study countries in Latin America, and Europe (Spain and Belgium): the type of participating actors and their roles, the main phases of the process, and the procedures for documenting the process.

The PAR process for the design and implementation of the interventions is led in each country by a local steering committee (LSC), which is a stable participation device made up of stakeholders of the intervention healthcare network (managers, professionals, users) nominated by the participating institutions, and the research team in the role of facilitators (capacity building, systematization, monitoring and feedback). The LSC is in charge of defining the strategy for returning results to the network, facilitating the creation of a working group called the professionals’ platform (PP) with those interested in taking action and, together with the PP, making the final selection and design of the interventions. In other words, the LSC has autonomy in deciding how to carry out its own process.

**Fig. 1:**
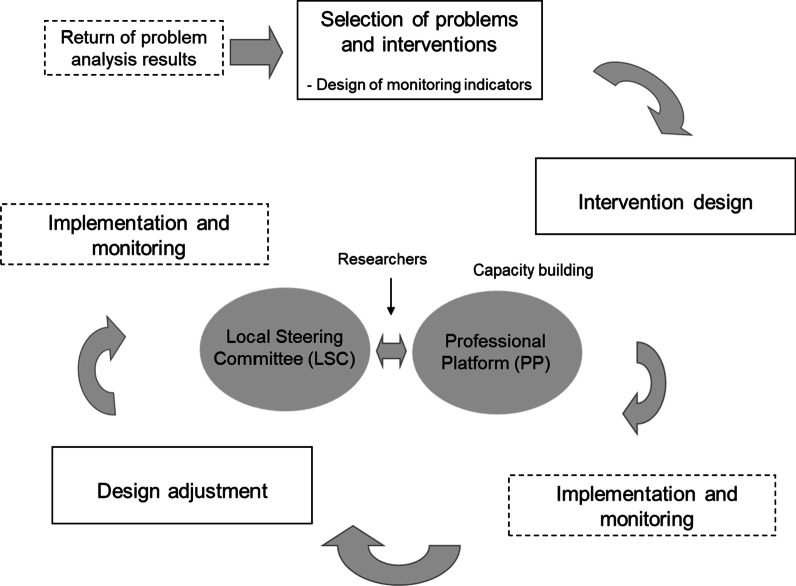
The participatory design process of the planned interventions

There are four main stages in the participatory design process (Fig. 1):

*1)* *Baseline* *study* *on* *cross-level* *care* *coordination* *and* *continuity* *in* *the* *healthcare* *networks,* utilizing qualitative and quantitative methods, to produce rigorous and valid evidence on cross-level care coordination and continuity problems in the study networks, their causes and consequences for care quality, as well as suggestions for improvement (actions), based on the experience of the main stakeholders (professionals, managers and users) [1, 35, 36].

*2)* *Return* *of* *results* *and* *formation* *of* *professionals’* *platform* *in* *the* *intervention* *network.* The baseline results are presented to professionals and managers of the intervention network in each country to carry out a collective, action-oriented analysis of care coordination and continuity problems. During the return of results, doctors and other professionals of the different care levels interested in participating (on a voluntary and non-remunerated basis) in the design and implementation of the intervention are also identified. These professionals make up the PP. To achieve sustained participation of PP members over time, it is recommended that agreements are established with network managers to free up time for participation and provide academic certification of the training given to its participants during the intervention.

*3)* *Selection* *of* *problems* *and* *interventions.* The PP, with the support of the LSC, first selects the problems through discussions guided by criteria (level of importance, potential for improvement, and feasibility of intervention) established through consensus techniques [37]. Interventions are then selected by the same method, taking into account suggestions made in the baseline study and the previous experience of the network and/or participants. The selection criteria include technical feasibility, short-term impact, long-term sustainability, and political visibility.

*4)* *Definition,* *planning* *and* *adaptation* *of* *the* *interventions*. For the selected interventions, the content, implementation strategy and monitoring plan are established. Following an initial implementation phase (approx. 6–8 months), a second PAR cycle is envisaged for collective reflection on the results of the monitoring, and identification of necessary adjustments to the design and implementation of the interventions.

Documentation of the participatory process followed in the design of the interventions is carried out in each country by two means: (1) keeping records of meeting minutes, agreements, activities, fieldnotes and any other document produced in the process; and (2) based on the former documents, comprehensive reports on the intervention design and implementation plans, implementation monitoring reports, and presentations made for the teams’ monthly teleconferences and biannual in-person meetings, as well as for public seminars, based on common templates to ensure the feasibility of the comparative analysis of the process.

The aim of this article is to analyse the participatory design process of the interventions to improve cross-level clinical coordination carried out in public healthcare networks of six Latin American countries.

## Methods

### Study design and study area/contexts

A descriptive qualitative study, based on document analysis, was carried out to analyse the participatory process of intervention design in each country. The study was carried out in the intervention healthcare network of each country: Argentina, north/north-western districts of Rosario; Brazil, the municipal network of Caruaru (Pernambuco); Chile, the northern network of Santiago; Colombia, south-western district network of Bogotá; Mexico, municipal network of Xalapa; Uruguay, three districts of the public network of the western region. The population covered varied from approximately 110,000 inhabitants in Argentina and Uruguay to 440,000 in Chile and Colombia, located in urban areas of large municipalities, except in Uruguay (smaller municipality) [38].

### Sample

All documents produced during the intervention design and implementation process in each country (November 2015–August 2018) describing the design and planning of the interventions, progress in the design process, and characteristics of the selected interventions were selected: reports on the design of interventions and implementation plans, monitoring reports, conferences/seminars presentations and meeting minutes (Table 1).

**Table 1:** Sample of documents analysed

	Argentina	Brazil	Chile	Colombia	Mexico	Uruguay
*Type* *of* *document*						
Reports on intervention design and implementation plans	4	6	2	3	5	1
Implementation monitoring reports	2	3	1	2	2	1
Conference presentations and meeting minutes	9	16	8	6	6	13
Total	15	25	11	11	13	15

### Data collection

Document analysis was conducted using a topic guide [39] to extract the data from the selected documents. The main dimensions included in the topic guide were: (1) characteristics of local steering committee and professionals’ platform (number and profile of participants, type of activities carried out); (2) intervention design process—phases (type, duration and number of PAR cycles), participants (number, profile and role) and number of sessions, methods applied; (3) difficulties encountered during the intervention design process; (4) problems selected and characteristics of the interventions designed (type of intervention, contents, methods, participants and monitoring (contents and method) proposed).

### Data analysis and quality of information

A thematic content analysis [39] was conducted. In the first stage, the design process in each country was analysed on a stand-alone basis, and in the second, a comparative cross-country analysis was performed. Data from the documents were segmented by design phase and themes. The analysis was conducted in the following stages: (1) reading and organisation of data; (2) identification of contents and generation of categories and subcategories; (3) description of results; (3) interpretation of results. The categories were generated from mixed sources: the main categories were taken from the topic guide and the subcategories were identified from the data. Themes that appeared in the documents were identified, coded, and re-coded, using an identifying code to return to the source document (data reduction). Subsequently, the data were classified through matrices of categories (data display). The creation of the categories was a dynamic process of going back and forth between the proposed categories and the data, in order to ensure that they made sense and were suitable for the subsequent classification of all data.

In order to ensure its quality, eight analysts (6 from each national research team and 2 from the international research team) participated in the analysis: the national analysts carried out the intra-country analysis and collaborated with the international analysts in the comparative analysis. The analysts had different backgrounds and a good knowledge of the subject and the context. Moreover, they were familiar with the documents to be analysed as they had been involved in facilitating the intervention design process and its documentation. Differences between analysts were discussed until a consensus was reached.

## Results

The analysis revealed differences in the participatory processes carried out in the six countries. The main findings are presented in relation to the characteristics and participation of the stakeholders, the intervention design process, and the characteristics of the designed interventions.

### 1. Characteristics and participation of local steering committees in the design of interventions

In all six countries a local steering committee (LSC) was established with healthcare network managers and the research team, with some also containing health professionals (in Brazil, Colombia and Chile) and user representatives (Brazil and Colombia) (Table 2). Its size ranged from 5 to 8 participants in Argentina, Colombia, Mexico and Uruguay, and 13 to 19 in Brazil and Chile*.* Membership remained stable during the intervention design process in Brazil and Uruguay, in Chile it grew as new centres joined the project, and there was considerable turnover in the rest. In Mexico and Colombia, the turnover was due to politically motivated changes in the network management team, as well as to a network restructuring process in Colombia, which led to the dismissal of professionals; and in Argentina it was due to changes in the research team. All the LSCs held monthly meetings to carry out programmed activities to design the interventions, except in Argentina, where the LSC was officially established but only certain members participated in the process, in collaboration with the professionals’ platform.

**Table 2:** Local steering committee and professionals’ platform characteristics by country

	Argentina	Brazil	Chile	Colombia	Mexico	Uruguay
*Local* *steering* *committee*						
Moment of formation	Baseline	Baseline	Baseline	Before baseline	Before baseline	Baseline
Participants (nº, profile and changes) *	7 participants – Managers and middle managers – No changes	13 participants – Managers; PC and SC professionals; user – No changes	19 participants Managers and middle managers; PC and SC professionals – Growth (8 to 19)	6–8 participants Managers and middle managers; PC and SC doctors; user -Absence of some managers	6 participants Managers and middle managers – Turnover of majority	5 participants Managers – No changes
Type of activities	– No meetings – Some participate in PP activities	– Monthly meetings – Dissemination strategy, composition of PP, selection of problems and interventions, design	– Monthly meetings – Dissemination strategy, composition of PP; final selection of problems and interventions; strategic management of design	– Monthly meetings – Dissemination strategy, composition of PP, final selection of problems and interventions, design	– Monthly meetings – Dissemination strategy, composition of PP, final selection of problems and interventions, design	– Meetings every two months – Dissemination strategy, composition of PP, preliminary and final selection of problems and interventions, design
*Professionals’* *platform*						
Moment of formation	Return of results	Return of results	After return of results	After return of results	After return of results	Before return of results
Participants (nº, profile and changes)	9 PC and 4 SC – Doctors, administrator, middle managers – No changes	16 PC and 10 SC – Doctors, other health professionals – No changes	8 PC and 9 SC – Doctors, other health professionals, administrators – Turnover 1 SC	17 PC and 6 SC – Doctors, other professionals – Turnover	19 PC and 12 SC (2 shifts) – Doctors, other health professionals, middle manager – No changes	6 PC and 9 SC – Doctors, other health professionals, middle manager – Turnover
Type of activities	Selection of problems and interventions, design of interventions	Selection, design and adjustments (some members)	Selection, monitoring of design	Selection (some members), design and adjustment (all)	Selection and design	Selection and design

*PC* primary care, *SC* secondary care, *PP* professionals’ platform

*Not counting the research team

### 2. Intervention design process

The participatory process for the intervention design was developed in two stages: (I) the return of results and selection of problems and interventions, and (II) the design and planning of interventions. Its development and the difficulties faced related to the context and the research teams differed according to country.

#### Return of results and selection of problems and interventions

Two types of process emerged: firstly, those in which the problematization and preliminary selection of the interventions were conducted first with professionals in each centre of the network, and the process was then finalized with the professionals’ platform and local steering committee (Brazil, Chile and Colombia); and secondly, those in which the selection of problems and interventions was only carried out with the professionals’ platform and local steering committee (Argentina, Mexico and Uruguay) (Table 3).

**Table 3:** Intervention design processes

	Argentina	Brazil	Chile	Colombia	Mexico	Uruguay
*Return* *of* *results*						
Time	After baseline	After baseline	After baseline	After baseline	After baseline	After selection
Size (nº centres and sessions)	5 sessions in each PC centre, 1 session in SC	3 sessions	24 sessions in each PC and SC centre	24 sessions in each PC and SC centre	11 sessions in each PC and SC centre	4 sessions
Participants (nº and profile)	83 participants Doctors, other health professionals, administrators, managers	59 participants Doctors, other health professionals, managers and users	479 participants Doctors, other health professionals, administrators, managers, users	200 participants Doctors, other health professionals, managers	280 participants Doctors, other health professionals, administrators, managers	65 participants Doctors, other health professionals and users
Dissemination method	-Presentation of results, followed by discussion – Results reports	– Presentation of results and preliminary selection – Reports and leaflets for managers, professionals, users	– Presentation of results and preliminary selection – Graphic materials and summary booklet	– Presentation of results and preliminary selection – Reports, leaflets and videos for professionals and patients	– Presentation of results, followed by discussion	– Presentation of results, followed by discussion – Summary booklet
*Selection* *of* *problems* *and* *interventions*						
Time	During return of results	During return of results	During return of results	During return of results	After return of results	Before return of results
Phases, nº participants* and sessions	Selection with PP (lead) and LSC – 18 participants, 2 sessions	1º) Preliminary selection: 28 participants, 3 sessions 2º) Selection with LSC (lead) and PP: 37 Participants, 4 sessions	1º) Preliminary selection: 479 participants, 24 sessions 2º) Selection with PP (lead) and LSC: 34 Participants, 5 sessions	1º) Preliminary selection: 200 participants, 24 sessions 2º) Selection with LSC (lead) and PP: 21 participants, 4 sessions	Selection with PP (lead) and LSC: 31 participants, 4 sessions	1º) Preliminary selection LSC:5 participants, 3 sessions 2º) Selection with LSC (lead) and PP: 20 participants, 10 sessions
Methods	– Guided group discussions	– Individual prioritization criteria and nominal group	– Guided group discussions	– Individual prioritization criteria and nominal group	– Prioritization criteria	– Individual prioritization criteria and nominal group
*Design* *and* *planning* *of* *interventions*						
Type of design and phases	– Open-ended design in phases, to be defined as phases are implemented (18 months) – Not all phases are implemented	– Design in 2 PAR cycles 1º) Initial design and planning (2 months) 2º) Adjustment following first implementation phase (5 months) – Some interventions and adjustments designed by the MHD	– Design in multiple PAR cycles (10 months) 1º) Formation of roundtables and work groups for design 2º) Pilot of main components 3º) Evaluation and adjustment	– Design in 2 PAR cycles 1º) Initial design and planning (2 months) 2º) Adjustment following first implementation phase (5 months)	– Design in 2 PAR cycles 1º) Initial design and planning (2 months) 2º) Adjustment following first implementation phase (5 months)	– Open-ended design, to be specified over the course of the intervention (7 months) – RT takes on activities assigned to PP
Method	Initial proposal of RT, discussed with PP, LSC and network managers	Proposal discussed with LSC	– PAR method: systematization of experience – collective reflection – action	– Initial proposal of RT, discussed with PP, LSC and managers	Proposal discussed with LSC and PP, and SHD (1º PAR cycle)	– Initial proposal of RT, discussed with PP and LSC
Participants (nº and profile)* and nº sessions	– PP and LSC – 31 participants, 34 sessions	– LSC – 22 participants, 7 sessions	– LSC (11 participants, 12 sessions) – Roundtables and groups: middle management and professionals of network (33 participants, 31 sessions) – Pilot tests: network professionals (90 participants, 4 sessions)	– PP and LSC – 26 participants, 5 sessions	– LSC and PP – 46 participants, 24 sessions	– PP and LSC - 20 participants, 4 sessions

*PC* primary care, *SC* secondary care, *MHD* Municipal Health Department, *LSC* Local steering committee, *PP* Professionals’ platform, *RT* research team, *SHD* State Health Department

*Not counting research team

##### Return of results and formation of professionals’ platform

The return of results was performed on a wider scale in Chile, Colombia and Mexico (in every centre of the network, ranging from 11 to 24 sessions, reaching 479 professionals in Chile) (Table 3). The profile of the participants was similar: professionals of different care levels and disciplines, managers, and in some cases also users. In all cases the same method was followed: presentation of the baseline study results and discussion with participants. For wider dissemination, in nearly all countries printed material was created and distributed (reports, leaflets).

During this phase, the professionals’ platform (PP) was formed with those who expressed an interest in participating (Table 2) and also—in Argentina, Colombia and Mexico—with some representatives appointed by the network’s management or local steering committee (LSC). In Uruguay, however, all the members were appointed. The PPs had approximately 15 participants of the different care levels (31 in Mexico, divided into two groups according to work shifts). These were mostly doctors, although there were also other health professionals and administrative personnel, and in some cases, middle managers. The number of participants from primary care slightly outnumbered those from secondary care, except for Chile and Uruguay, where this was more balanced. In Argentina there was a considerable turnover in members, as some were assigned managerial duties, and some new members joined to improve operations. In Brazil, the low level of participation in the PP sparked its fusion with the LSC.

##### Selection of problems and interventions

Depending on the country, those in charge of leading the selection of problems and interventions were the PP, supported by the LSC (Argentina, Chile and Mexico), or the LSC together with some PP members (Colombia, Brazil and Uruguay) (Table 3). In most countries this was carried out separately in successive sessions (first problems then interventions). In Chile, Colombia and Brazil the preliminary selection made at the return of results phase was used as a springboard for the discussions. In all cases, the final selection was made in a joint session of the LSC and PP. The total number of meetings with the PP and/or LSC varied from 2 (Argentina) to 6 (Uruguay).

I) *Preliminary* *selection* *of* *problems* *and* *interventions* (Chile, Colombia, Brazil). Following the presentation of results, the nominal group technique was used to identify the most important problems of cross-level care coordination. Causes, consequences and feasible interventions were then analysed until a matrix of problems and intervention proposals was obtained (Table 3).

II) *Selection* *of* *problems*. Problem selection began with a presentation of the problems selected in the return of results phase in Chile and Brazil, with the baseline results in Argentina, Mexico and Uruguay, and with both in Colombia (Table 3). The final selection was based on defined criteria which, in Mexico and Uruguay, were applied using a prioritization matrix, and in all the other countries, served to steer the group discussions or nominal groups.

III) *Final* *selection* *of* *interventions*. In all cases, interventions proposed by professionals in the baseline study were taken into account, as were interventions implemented in other contexts. In Chile and Colombia, interventions selected in the return of results phase and previous experiences in the network were also taken into consideration. While in Uruguay and Colombia a prioritization matrix was used for the final selection, in the rest of the countries it was carried out through discussions that addressed the criteria defined during the planning of the process. In Brazil, however, political feasibility (in the pre-electoral context) and thus the work of the Municipal Health Department in diabetes care were also factored in (an aspect not contemplated by the PP). Furthermore, the intervention initially selected in Mexico (online communication system) did not feature on the prioritized list made by the platform either.

#### Design and planning of interventions

Three types of experiences in the design and planning of interventions can be distinguished, according to their duration or dynamics (Table 3):

1. Short design and planning process (3 months), with adjustment after the first implementation phase (5 months), in Colombia, Brazil (approx. 5–7 meetings and 22–26 participants) and Mexico (24 meetings and 46 participants). The final design was produced with the PP/LSC.
2. Longer and more participatory process in Chile (10 months), in which the design and planning of the intervention were progressively defined over successive cycles of action/reflection that included pilot tests of the main components and the broad participation of stakeholders (approx. 47 meetings and 134 participants), divided into three groups with different roles: i) roundtables (working subgroups) in charge of design, with the participation of middle management and professionals related to the interventions, to which new stakeholders were added according to need; ii) professionals that participated in the pilots; and iii) LSC in a role of support, analysis and strategic decision-making on the intervention.
3. An open-ended process was attempted in Argentina and Uruguay, in which the components were gradually and collectively defined with the PP over the course of the different phases. However, in Argentina, the process took longer than anticipated (18 months, 34 sessions and 31 participants) and as a result, some of the phases could not be implemented. In Uruguay, the research team had to take on design activities due to operational problems with the professional’s platform (7 months, 4 meetings and 20 participants).

#### Difficulties encountered during the intervention design process

The main problem identified in the design process, to a lesser extent in Chile, was the limited participation of SC doctors—and in Colombia and Uruguay, also of primary care professionals and managers—which limited the return of results and functioning of the PP (Table 3). This was attributed to difficulties in finding time to attend the meetings, despite agreements with the networks for institutional support to free up professionals' time. In Uruguay, the distance between centres of the network (and thus PP members), and between the centres and the research team, also influenced operations, although attempts were made to mitigate this through communication online. Lastly, the electoral political climate hindered LSC operations in Brazil, Mexico and Colombia, and PP operations in Argentina, which led to delays in activities because of the complications involved in arranging meetings and a lack of commitment to the initial agreements made due to the imminent turnover of members. The change of government led to the replacement of some LSC members in Brazil and Mexico. In Argentina, changes in the research team during the design phase also contributed to delays in activities.

### Problems selected and characteristics of the designed interventions

#### Problems selected

The main problem selected in all countries was a lack of communication between general and specialist doctors regarding the care of patients, along with limited cross-level exchange of clinical information in Argentina, Brazil and Mexico, and disagreement over the clinical management of patients across care levels in Brazil, Colombia and Mexico. Other problems selected were waiting times for SC in Argentina, and a low level of trust and collaboration between levels and the lack of a common vision in the Chilean network.

#### Characteristics of the designed interventions

To address the selected problems, three main types of intervention were chosen: (i) joint meetings between primary and secondary care doctors for the discussion of clinical cases, and/or ongoing training in Brazil, Colombia, and Chile; (ii) online (asynchronous) inter-level consultations to resolve queries on patient management and referral in Brazil and Mexico, which envisaged the subsequent development of joint training based on the consultations made; and (iii) the creation and implementation of shared care guidelines in Argentina and Brazil. Two further interventions were selected that did not fall into the above categories: in Uruguay, a strategy to promote the use of the referral and counter-referral form, and in Chile, in addition to the above intervention, an induction program for working in the network to promote integration into the organizational culture of the network (see Others in Table 4). The adjustment process led by the LSC after the first implementation phase resulted in the interventions in Brazil being extended to include mental health professionals, and in the development of joint training sessions in Mexico. Table 4 shows the main characteristics of the interventions.

**Table 4:** Characteristics of the designed interventions by type and country

Joint meetings	Brazil Joint discussion of clinical cases in diabetes and mental health	Chile Joint virtual clinical conferences	Colombia Joint meetings for discussion of clinical cases and medical training	Mexico Joint training sessions
Participants	– Doctors and other health professionals from PC and psychiatrists and endocrinologists of the network	– Doctors and other professionals from PC and SC in the network	– PC and SC doctors *(PP)* and other PC doctors *(replica* *sessions)* and other professionals of the network	– PC and specialists of the network
Characteristics	– Discussion of clinical cases (diabetes and mental health) – In person, monthly, duration 4 h	– Discussion of cases, referral criteria and follow-up instructions (any pathology) – Online (platform), fortnightly, duration 2 h	– Discussion of cases, referral criteria and ongoing training (chronic diseases) – In person, monthly/every two months, duration 4/2 h according to the type	– Ongoing training, discussion of cases (maternal-perinatal and chronic) – In person, sporadic, 3 days, 8 h
Monitoring (aspects, method)	– participation, satisfaction functioning – indicators and questionnaire for participants	– participation, usefulness, functioning, aspects for improvement – indicators, questionnaire and observation of non-participants	– participation, usefulness, functioning, aspects for improvement – indicators, focus groups and observation of non-participants	– participation – indicators

Offline virtual consultations	Brazil Virtual consultations between levels	Mexico Virtual communication system between levels
Participants	– Interested PC doctors, endocrinologists and psychiatrists	– Interested PC and SC doctors
Characteristics	**– **asynchronous inter-level consultations for endocrinopathies and mental health – email, standardized forms – maximum response time: 8 days	– asynchronous inter-level consultations, agreements on referral criteria and protocol repository, in chronic diseases and maternal health – digital platform hosted on server, standardized forms – maximum response time: 3 days
Dissemination and training	– Bulletins to centres, MHD webpage, dissemination in meetings – No training plan developed	– Active search for participants in each health centre – Training plan and users’ manual
Monitoring (aspects, method)	– utilization, response time, barriers to use, dissemination and training – indicators, questionnaire	– utilization, response time, barriers to use – indicators, focus groups

Shared care guidelines	Argentina Care agreement for patients with hypertension and diabetes	Brazil Diabetes shared care guidelines
Participants	Selected PC and SC doctors, PP and management representatives (creation phase)	Doctors and other health professionals from PC and endocrinologists, and management representatives (creation phase)
Phases	I) Creation: 1) characterization of care path; 2) joint meetings for clinical cases, 3) meetings to draw up guidelines II) Implementation	I) Creation: 1) joint meetings for clinical cases, 2) meetings to draw up guidelines II) Implementation
Dissemination and training	– No dissemination or training plan developed	– dissemination in training meetings and material sent to units – group training sessions
Monitoring (aspects, method)	– participation in creation – indicators	– participation in creation and training sessions, satisfaction with training sessions, knowledge and use of guidelines – indicators and questionnaires

Others	Uruguay Strategy to promote use of referral and reply letter	Chile Induction program for working in the network
Participants	– PC y SC doctors, PP (in charge of operations in each centre)	– Professionals of both care levels, but focusing on those starting to work in the healthcare network
Characteristics	– Standardized forms, flow chart and regulations of use	– Bidirectional inter-level visits between PC and SC: in groups according to profile – Graphic and audio-visual informative dossier on the network. Intranet for dissemination of material – Pending creation of implementation plan for the program
Dissemination and training	– Plan for implementation in healthcare units: delivery of material, dissemination and monitoring	
Monitoring (aspects, method)	– utilization, quality of records, barriers and facilitators in implementation – indicators, observation and focus groups	– level of participation, usefulness, functioning, aspects for improvement – indicators, questionnaire and observation of non-participants

*PC* primary care, *SC* secondary care, *PP* professionals’ platform, *MHD* Municipal Health Department

- a) Joint meetings between care levels

In-person joint meetings were designed in Colombia and Brazil, and also in Mexico following the adjustment, and online meetings were designed in Chile (Table 4). These addressed issues such as referral criteria, care agreements and patient follow-up instructions. The focus in Colombia and Brazil was on chronic diseases, in Mexico on maternal and child health care, and in Chile on any disease, according to professionals’ needs. They were aimed at doctors of the network and, to a lesser degree, at other professionals, with the exception of Chile where they were multidisciplinary. They were scheduled periodically, except in Mexico, and took a participatory approach centred on discussing and resolving cases jointly, with the moderation of a facilitator (generally the specialist).

- b) Offline virtual consultations

Offline virtual consultations between primary and secondary care doctors were designed for selected pathologies, using standard forms sent through a digital platform in Mexico and institutional email in Brazil (Table 4). In Mexico, the intervention also factored in the use of the digital platform as a repository of official Mexican regulations and clinical practice guidelines related to the selected pathologies, and later, the organization of joint training meetings on the most frequent topics of consultation.

- c) Shared care guidelines

In Argentina and Brazil, interventions were selected for the creation and implementation of shared primary and secondary care clinical practice guidelines for certain chronic diseases, which stipulated both the care procedures and administrative pathways required to deliver care to patients. In both countries, the guidelines were drawn up on the basis of meetings between primary and secondary care to discuss clinical cases, with the subsequent incorporation of representatives from the management of the network.

- d) Strategy to promote the use of the referral and reply letter (Uruguay)

This was a three-part strategy: creation of a standardized referral and counter-referral form, a flow chart on the use of the form in the network, and regulations with instructions for use and responsibilities. PP members were in charge of the implementation of the intervention in their respective establishments (preparation, information and monitoring).

- e) Induction program for working in the network (Chile)

This was a network-focused work induction strategy directed at all professionals but focusing on those starting work in any of the network’s centres. It consisted of two parts: two-way inter-level visits between primary and secondary care teams, for professionals to get to know each other, build contacts and experience the work climate of the other level, and a dossier with graphic and audio-visual material providing information on the teams and activities of the network. The network coordinator—the Northern Metropolitan Health Service (SMMN)—acts as program administrator and those in charge of in-centre induction activities are responsible for putting it into practice.

## Discussion

Despite the increasing insistence of experts that health professionals should play a part in the selection and design of health services interventions, there are few studies available to date on the use of participatory design processes, especially for interventions to improve cross-level care coordination. The results of this study, based on experiences in six different Latin American care settings, offer valuable information for the formulation of strategies and national and local policies on the critical aspects of participatory designs in health services interventions, as well as guidance on the type of interventions that are most relevant to health professionals in the improvement of clinical coordination.

1. Differences in the participatory process of design across settings and over time

As expected in PAR, the development of the participatory process differed across countries and over time in aspects such as levels of participation, the PAR cycles developed and the type of collaboration between researchers, managers and professionals. The level of participation in the LSC, PP and working groups was higher in Chile, and in Colombia too in the initial intervention selection phase. However, it was only in Chile that participation grew over time, while in all other countries, problems were encountered in maintaining participation levels. The process in Colombia, Mexico and Brazil was carried out in two PAR cycles, with a short planning and design phase previous to the implementation phase, while in Chile it was longer, with multiple iterative cycles of action/reflection in which the implementation and evaluation of some components (pilots) formed part of the design. This meant that the process in Chile was more flexible, progressive and dynamic, although in the other countries there were some open contents and methods (i.e., with no initial planning), so that they could be gradually defined or altered as the interventions were being implemented. Uruguay and Argentina adopted an open-ended design process; however, difficulties encountered in implementation, especially in Argentina, meant that some elements of the design and planning of the interventions did not come to fruition or were conducted in a less participatory way. Lastly, the type of collaboration established during the design of the interventions [31] was cooperative in most countries: LSC and PP worked together with researchers in the design of the interventions, but responsibility for directing the process remained with the researchers; except in Chile, where the collaboration was more of a co-learning type: LSC, PP, working groups and researchers shared their knowledge to create new understanding and worked together to develop action plans, and the researchers acted as facilitators.

The kind of design process adopted in Chile—growing participation, multiple PAR cycles, flexible co-learning-based collaboration—is considered the most appropriate in the sense that the resulting interventions achieve greater social validity, uptake and impact on care practice, penetration, and sustainability over time [18, 21, 22]. The studies carried out on the perceived outcomes of the interventions [32] and the PAR process in this study [31] from the stakeholders’ perspective also found that this kind of design, especially for the virtual inter-level consultations in Chile, was associated with better results in the uptake and sustainability over time of the interventions.

However, the results also show, as Greenwood [40] and others authors have argued, that this kind of design is not possible in every situation, and that the scope of PAR will vary over the course of the process depending on the context, knowledge and skills of the participants (especially of the research team), the nature of the problem and the aims and contents of the intervention [15, 18, 22]. In the experiences analysed here, considerable contextual difficulties were encountered during the participatory design process: firstly, the limited institutional support given to allocating working time of professionals, particularly specialists, to participate in the intervention design and planning meetings; and secondly, the interference of politics and changes in management that led to a turnover in LSC and PP members and hampered the committees’ operations during the process [31]. Discontinuity in doctors’ participation was also related to inadequate working conditions (temporary and/or part-time contracting) in some contexts[31]. The turnover of participants complicates the PAR process because it requires repeated cycles of action/reflection, over the course of which new knowledge is progressively built, and a climate of shared responsibility, collaboration and trust between researchers and practitioners is gradually created [20].

The analysis of the design process also revealed difficulties related to the role of the research team, such as the change in its members in Argentina, which paralyzed activities, or the bias it displayed towards the selection of one of the interventions in Mexico, contributing to a lack of interest in its adoption among professionals [41]. In this kind of scenario, bottom-up designs with shorter, more closely planned PAR cycles, such as those carried out in Colombia or Brazil, might be more suitable.

2. Mechanisms based on mutual feedback: key to improving clinical coordination

Despite the PAR process being carried out in different contexts, the health professionals involved coincided in their selection of coordination problems to address (lack of direct communication in patient follow-up and limited clinical agreement) and the type of interventions to implement. Although the type varied—joint meetings to discuss cases, creation of shared care guidelines or induction programs—they shared common characteristics: they were based on mutual feedback between professionals, they were aimed at professionals of the different care levels in the network, and they included elements of participatory learning methods based on reflecting on one’s own practice and joint decision-making. Feedback—as a way to coordinate activities—is based on the exchange of information and direct communication between the professionals involved, so that the level in which the information is generated is able to solve the problem [4, 6]. It is recommended by the theory of organizations [4, 6] in circumstances of high uncertainty and interdependency in which standardization is not possible. Participatory learning methods have proved effective in changing and improving clinical practice, as long as certain elements are guaranteed [42]: participant interaction that establishes an egalitarian dialogue in which everyone can reflect on their own experience of clinical practice and build agreements together, work in small groups, and the appointment of a facilitator—a known leader respected by participants—to participate throughout the process. These aspects were shown in the evaluations of the intervention implementation process to be determinant in promoting change in clinical practice [38, 41, 43]

These are, therefore, strategies that are simple to put in place, requiring a low input of resources (mainly professionals’ time), but multifaceted. In other words, they do not just address the problem—lack of clinical agreement—but also, on being based on direct feedback and participatory learning methods, help to improve factors that influence coordination and the low uptake of mechanisms, such as lack of trust or willingness to collaborate with the other care level [1, 38, 44, 45]. These aspects, which have been highlighted by various authors [46, 47] as key to fostering intervention uptake and permeating clinical practice, may be even more relevant in settings with a greater scarcity of resources.

### Limitations of the study

This study is based on the analysis of documents produced during the intervention design and implementation process in each country, which could pose several limitations. It could limit the scope and depth of the analysis, in the sense that there might be aspects of the process that were not fully documented. However, in the cases in which the reports generated did not provide sufficient information on the process of designing the interventions, the presentations and meeting minutes on the progress of the process were analysed.

Furthermore, although the documents are descriptive and were produced, divulged and discussed with the different stakeholders (LSC, PP, etc.), they may reflect the perspective of the researchers more intensely. This limitation was addressed by taking into consideration, in the interpretation and discussion of results, previously published data from the qualitative study on the intervention implementation process from the different participants’ viewpoint [22, 23].

## Conclusions and recommendations

The results of this study show that PAR processes can be used to identify problems of clinical coordination across care levels and design interventions that are sustainable and adequate to the needs and resources of the context. It is often argued that the results of this type of process are of purely local validity, and that the only part that is replicable in other contexts is the participatory method. Nevertheless, some lessons can be drawn from the experiences analysed in this study for stakeholders who wish to implement wide-scope participatory processes for the design or adaptation-to-context of interventions, strategies and policies to improve cross-level care coordination: (1) A minimum of suitable contextual conditions: time to participate and institutional stability at the level of technical staff and professionals. In contexts of high uncertainty or adverse conditions (e.g. in which political transitions translate into changes in management teams, in political priorities, and thus also in institutional support for interventions), it is preferable to have shorter participatory processes, with a lower level of participation, but flexible planning (that gives careful consideration to the elements of the interventions that are subject to potential modification, through collective reflection throughout the process) and broad dissemination of the interventions. In these contexts, flexibility of design is also key, allowing interventions to be adapted to new institutional objectives, as well as the inclusion in the LSC of middle managers, who are less influenced by political changes; (2) Time and a gradual pace, with a limited initial scope that is progressively expanded, and voluntary in character. Time is needed to establish groups, processes, and relationships and to generate interest among professionals and managers to secure institutional support, so that the participatory process can permeate the organization. This is especially true in the case of interventions to improve coordination in healthcare networks that also need to address lack of trust, knowledge and poor communication between actors of different care levels. Moreover, for the process to be sustainable over time, participation must not be imposed, but rather generated voluntarily among interested parties; (3) Good preparation of the PAR process. This requires, firstly, an analysis of conditions to implement the process in the local context, identifying barriers and defining strategies to overcome them (these should include the establishment of institutional agreements for the creation of the LSC and working groups, protection of the participants’ time, and the involvement/awareness of managers); and secondly, the definition of roles, type of participants, time and resources, taking into consideration the efforts required to install the PAR process in the organization. However, the planning of the PAR process must at the same time be flexible, so that it can be adapted to any changes that occur during the process, and must be carried out in collaboration with the stakeholders; (4) Utilization of participatory methods, specifically, fostering spaces for feedback between professionals of different care levels, adequate analysis of the problems and reflection on own practice, flexibility in the adaptation of intervention content, horizontal and democratic participation, keeping to agreements jointly made, and the presence of a facilitator. Participatory methods are applicable throughout the whole process (design, planning, implementation, and evaluation) and for any type of intervention to improve care coordination.

## Data Availability

Most of the documents which constitute the dataset necessary to replicate our study is publicly available in the repository located in a specific section of the project website (https://www2.equity-la.eu/en/publicaciones.php?t=IS). For further information, please contact the corresponding author (ivargas@consorci.org).

## Abbreviations

PAR: Participatory action research
LSC: Local steering committee
PP: Professionals’ platform
PC: Primary care
SC: Secondary care
LA: Latin America
